# Clinical therapeutic effects of pulsed-radiofrequency combined with blockade in the treatment of patients with chronic migraine: A protocol for systematic review and meta-analysis

**DOI:** 10.1097/MD.0000000000027698

**Published:** 2021-11-12

**Authors:** Guang-Zhi Zhang, Yao Chen

**Affiliations:** a Department of Pain Management, People's Hospital of Dongxihu District, Wuhan, Hubei, China; b Edong Healthcare City Hospital of Traditional Chinese Medicine (Infectious Disease Hospital), Ezhou, Hubei, China.

**Keywords:** blockade, chronic migraine, meta, pulsed-radiofrequency

## Abstract

**Background:**

Recently, researchers have emphasised on the clinical therapeutic effects of pulsed-radiofrequency combined with blockade to treat chronic migraine (CM) patients. However, there are controversial results. Therefore, the authors conduct the proposed research to assess the efficacy of pulsed-radiofrequency combined with blockade to teat CM patients.

**Methods:**

The authors will perform a comprehensive literature search on the following online-based databases from establishment till October 2021: Web of Science, EMBASE, PubMed, China National Knowledge Infrastructure, the Cochrane library, and WanFang database. We will consider all randomized controlled trials of pulsed-radiofrequency combined with blockade for CM for inclusion. There won’t be any language constraints. Following the search, a pair of reviewers will independently screen and choose related articles to include in the meta-analysis. The Cochrane risk of bias tool will be used to assess the systematic value of all included randomized controlled trials. The study will utilize the risk ratio, mean differences, or standardized mean differences and their 95% confidence intervals to perform an estimation of the pooled mean effect size. Lastly, the authors will employ funnel plot, Egger test, and sensitivity analysis to determine and describe possible heterogeneity.

**Results:**

The authors will publish the results in a peer-reviewed journal.

**Conclusion:**

The proposed study will be the first to evaluate the effectiveness of pulsed-radiofrequency combined with blockade in the treatment of patients with CM.

**Ethics and dissemination:**

Since the proposed study is a systematic review of published studies, an ethics approval is not needed.

**Registration number:**

Oct 12, 2021.osf.io/d2wx3. (https://osf.io/d2wx3/).

## 1 Introduction

Migraine is the second most prevalent condition that leads to disability. It diminishes the life standard, strains the economy, and diminishes productivity.^[1,2]^ In view of the widespread incidence of over 12% and the fact that migraine progresses throughout the most productive years in a person's life, there has been an urgency in the medical and pharmaceutical fields to study migraine.^[3,4]^ For most individuals, abortively treating the severe symptoms using triptans or simple analgesics is ineffective to relieve the harmful effects of the disease, particularly because drugs have little effect in at least 30% of migraine attacks. Besides, people have poor tolerance towards the drugs and overuse may even worsen the migraine. In addition to ineffective acute drugs, the decision to commence preventive therapy is taken physicians based on the frequency of attacks, days with headache, severity of attacks, and impact on the patient's life standard. Accordingly, over a third of the participants are suitable for prophylactic treatment.^[5–7]^

Chronic migraine (CM) is an acute form of migraine attack.^[8]^ As per the International Headache Society, CM is defined as a headache that occurs on 15 or more days in the space of 1 month for over 3 months annually. In other words, on a minimum of 8 days in 1 month with the characteristics of migraine without or with aura.^[9]^ Among the general population, the incidence of CM is between 1.4% and 2.2%, which shows that 8% of all individuals suffer from migraine.^[10]^ In most cases, CM evolves from episodic migraine, with a conversion rate of 2.5% to 3% annually.^[11]^ Reportedly, pulsed-radiofrequency has an obvious effect on preventing CM. However, the clinical therapeutic effects of pulsed-radiofrequency combined with blockade to treat CM patients remain controversial. This study aims to evaluate the effectiveness of pulsed-radiofrequency combined with blockade in the treatment of patients with CM.

## 2 Objective

This meta-analysis will be conducted to summarize previous studies to evaluate the clinical therapeutic effects of pulsed-radiofrequency combined with blockade to treat CM patients.

## 3 Methods

All reporting of the present study is in accordance with the preferred reporting items for systematic and meta-analysis protocols statement. Further, the study has been registered under the Open Science Framework (October 12, 2021.osf.io/d2wx3).^[12]^

### 3.1 Study selection criteria

#### 3.1.1 Study types

This metanalysis only considers randomized controlled trials (RCTs) related to the use of pulsed-radiofrequency combined with blockade for CM. Other types of studies shall be excluded, including reviews, case reports, conferences, and letters.

#### 3.1.2 Types of participants

The metanalysis will include participants confirmed to be diagnosed with CM. There is no restriction on age, gender, region, degree of illness.

#### 3.1.3 Types of interventions

The interventions of the empirical set will comprise pulsed-radiofrequency combined with blockade. The articles will be left out if the content includes pulsed-radiofrequency only, blockade only, or other treatments. The control group's interventions can include any treatments other than pulsed-radiofrequency combined with blockade.

#### 3.1.4 Types of outcomes

The primary outcomes are visual analogue scale, self-rating depression scale, self-rating anxiety scale, and the World Health Organization quality of life brief version. The secondary outcome includes adverse events.

### 3.2 Search methods for the identification of studies

#### 3.2.1 Electronic searches

The authors will perform a comprehensive literature search on the following online-based databases from establishment till October 2021: Web of Science, EMBASE, PubMed, China National Knowledge Infrastructure, the Cochrane library, and WanFang database. All RCTs of pulsed-radiofrequency combined with blockade for CM shall be selected to assess eligibility without any language restrictions. The following keywords will be used to perform the search: pulsed-radiofrequency, blockade, migraine, and RCTs.

#### 3.2.2 Searching other resources

The authors will perform a manual search in the reference lists of the retrieved literature to seek all potential grey literature. Moreover, the authors will also search Google Scholar to not miss out on any related article published online.

### 3.3 Data collection and analysis

#### 3.3.1 Study selection

The titles and abstracts of all potential studies will be assessed by two authors independently. The screening process will adhere to the established criteria with a standardized form. The procedure of selecting studies will adhere to the PRISMA flow chart. In the case of any disagreement, the authors will discuss and resolve the issue.

#### 3.3.2 Data extraction and management

Data extraction will be carried out using a form, where relevant data that satisfy the inclusion criteria will be gathered for analysis. Accordingly, a couple of reviewers will perform data extraction independently. Once the review concludes, the extracted data shall be compared. All issues shall be resolved through discussion. The extracted information will include name(s) of authors, date of publication, design of study, intervention method, study population, age, and outcome measures.

#### 3.3.3 Assessment of risk of bias in included studies

The Cochrane bias risk evaluation tool will be used to perform a methodological quality evaluation of all included RCTs. The bias risk tool comprises seven items, and the assessment of each item is categorized as low bias risk, unclear bias risk, and high bias risk. A pair of autonomous reviewers will assess the bias risk associated with each included article. The two authors will hold discussions to resolve any diversions.

#### 3.3.4 Measurement of treatment effect

In this study, continuous outcome data shall be represented in the form of mean differences or standardized mean differences with 95% confidence intervals. Moreover, dichotomous outcome data shall be represented as risk ratios with 95% confidence intervals.

#### 3.3.5 Dealing with missing data

In case there is insufficient data, unclear results, or even incomplete data, the authors will attempt to contact the original authors to request the data. Still, if there is no chance to obtain the relevant data, the authors will analyse the available data. Later, the authors will discuss the potential impact due to the limitation of missing the data.

#### 3.3.6 Sensitivity analysis

A sensitivity analysis will be conducted to detect the robustness and stability of pooled results, data, and systematic value.

#### 3.3.7 Assessment of publication bias

If the analysis includes over 10 studies, the authors will employ funnel plots to detect and define potential publication bias. Accordingly, Egger test will be preferred to detect funnel plot asymmetry.

## 4 Discussion

The proposed protocol for a systematic review will summarize the most recent data to evaluate the efficacy of pulsed-radiofrequency combined with blockade to treat CM patients. The study findings will present reliable, comprehensive, and updated evidence to determine whether pulsed-radiofrequency combined with blockade shows potential. Further, the outcomes will also present a reliable reference to implement CM treatment and collect data related to patients for both scholars and clinical practitioners, as well as the health policymakers.

## Author contributions

**Conceptualization:** Guang-Zhi Zhang, Yao Chen.

**Data curation:** Guang-Zhi Zhang, Yao Chen.

**Formal analysis:** Guang-Zhi Zhang, Yao Chen.

**Funding acquisition:** Guang-Zhi Zhang, Yao Chen.

**Investigation:** Yao Chen.

**Methodology:** Guang-Zhi Zhang, Yao Chen.

**Project administration:** Guang-Zhi Zhang, Yao Chen.

**Resources:** Guang-Zhi Zhang.

**Software:** Yao Chen.

**Supervision:** Guang-Zhi Zhang, Yao Chen.

**Validation:** Guang-Zhi Zhang.

**Visualization:** Guang-Zhi Zhang, Yao Chen.

**Writing – original draft:** Guang-Zhi Zhang.

**Writing – review & editing:** Yao Chen.
